# Post Hoc Bias in Treatment Decisions

**DOI:** 10.1001/jamanetworkopen.2024.31123

**Published:** 2024-09-04

**Authors:** Donald A. Redelmeier, Eldar Shafir

**Affiliations:** 1 Department of Medicine, University of Toronto, Toronto, Ontario, Canada; 2 Evaluative Clinical Sciences Program, Sunnybrook Research Institute, Sunnybrook Health Sciences Centre, Toronto, Ontario, Canada; 3 Institute for Clinical Evaluative Sciences, Sunnybrook Health Sciences Centre, Toronto, Ontario, Canada; 4 Division of General Internal Medicine, Sunnybrook Health Sciences Centre, Toronto, Ontario, Canada; 5 Center for Leading Injury Prevention Practice Education & Research, Sunnybrook Health Sciences Centre, Toronto, Ontario, Canada; 6 Department of Psychology, Princeton University, Princeton, New Jersey; 7 Princeton School of Public and International Affairs, Princeton, New Jersey

## Abstract

**Importance:**

A goal of health care is to reduce symptoms and improve health status, whereas continuing dubious treatments can contribute to complacency, discourage the search for alternatives, and lead to shortfalls in care.

**Objective:**

To test a potential bias in intuitive reasoning following a marginal improvement in symptoms after a dubious treatment (post hoc bias).

**Design, Setting, and Participants:**

Surveys eliciting treatment recommendations for hypothetical patients were sent to community members throughout North America recruited via an online survey platform in the early winter months of 2023 and 2024 and presented to health care professionals (pharmacists who were approached in person using a secret shopper technique) in the summer months of 2023.

**Exposure:**

Respondents received randomized versions of surveys that differed according to whether vague symptoms improved or remained unchanged after a dubious treatment.

**Main Outcomes and Measures:**

The primary outcome was a recommendation to continue treatment.

**Results:**

In total, 1497 community members (mean [SD] age, 38.1 [12.5] years; 663 female [55.3%]) and 100 health care professionals were contacted. The first scenario described a patient with a sore throat who took unprescribed antibiotics; respondents were more likely to continue antibiotics after initial treatment if there was a marginal improvement in symptoms vs when symptoms remained unchanged (67 of 150 respondents [45%] vs 25 of respondents [17%]; odds ratio [OR], 3.98 [95% CI, 2.33-6.78]; *P* < .001). Another scenario described a patient with wrist pain who wore a copper bracelet; respondents were more likely to continue wearing the copper bracelet after initial care was followed by a marginal improvement in symptoms vs when symptoms remained unchanged (78 of 100 respondents [78%] vs 25 of 99 respondents [25%]; OR, 16.19 [95% CI, 5.32-19.52]; *P* < .001). A third scenario described a patient with fatigue who took unprescribed vitamin B_12_; respondents were more likely to continue taking vitamin B_12_ when initial treatment was followed by a marginal improvement in symptoms vs when symptoms remained unchanged (80 of 100 respondents [80%] vs 33 of 100 respondents [33%]; OR, 7.91 [95% CI, 4.18-14.97]; *P* < .001). Four further scenarios involving dubious treatments found similar results, including when tested on health care professionals.

**Conclusions and Relevance:**

In this study of clinical scenarios, a marginal improvement in symptoms led patients to continue a dubious and sometimes costly treatment, suggesting that clinicians should caution patients against post hoc bias.

## Key Points

**Question:**

Do judgments about health treatments show a post hoc bias?

**Findings:**

In this survey study using a series of randomized scenarios, among 1497 patients and 100 clinicians, participants were more likely to continue (or recommend continuing) a dubious treatment when initial care was followed by a marginal improvement in symptoms, despite the change being a potential coincidence.

**Meaning:**

These findings suggest that intuitive reasoning in health care is prone to the post hoc bias, highlighting the importance of clinicians cautioning patients to avoid this bias.

## Teaser

This survey study examines responses from community members and pharmacists for a potential bias in intuitive reasoning (post hoc bias) when asked for recommendations regarding a hypothetical scenario describing a patient who experiences marginal improvement in symptoms after a dubious treatment.

## Introduction

The post hoc bias (also termed *post hoc ergo propter hoc fallacy*) has been recognized for centuries with enduring relevance. The general implication for medical care is that a patient who improved after a treatment did not necessarily improve as a result of the treatment.^1^ Correctly interpreting a marginal improvement in symptoms requires considering alternative explanations, such as withdrawal from adverse activity, added rest, physiologic homeostasis, adaptive immunity, the placebo effect, simple chance, or other confounders.^2^ Attributing an improvement to a treatment, however, is reassuring to patients, easily explained to families, and readily justified to others.^3^ Attributing an improvement to a treatment is also quick, common, intuitive, and gratifying.

Modern medical care involves fundamental uncertainty because disease mechanisms and treatment pathways are incompletely understood. The uncertainty also contributes to a trial-and-error style of reasoning in practice. The result is that patients and clinicians encounter abundant opportunities for post hoc bias when following up on treatment.^4^ For example, vitamin B_12_ supplements are sometimes started for vague symptoms, yet improvements are often marginal and some patients are misdiagnosed, leading to delayed diagnosis of a serious underlying disease that subsequently turns incurable.^5^ The uncertainty can also contribute to premature closure, polypharmacy, high costs, practice pattern variations, overtreatment, and ultimate disappointment.^6^

A tendency toward post hoc bias is similar to tunnel vision or status quo reasoning and can contribute to medical error.^7^ Despite the pitfalls of biased post hoc reasoning, however, direct tests of this rudimentary error in intuitive judgment are missing in evidence-based medicine and are rarely the topic of psychological research (beyond lectures or editorials).^8,9^ Here, we present a series of randomized surveys to test post hoc bias across diverse clinical cases that require subjective judgments. The hypothesis is that people are more likely to continue a treatment, however dubious, when initial care is followed by a marginal symptomatic improvement. Here, we test whether intuitive judgments about health treatments are prone to this bias.

## Methods

### Overview

For this survey study, we developed materials using methods adapted from large-scale behavioral decision science.^10^ This involved creating different case scenarios describing an individual patient with vague symptoms of ambiguous severity and additional relevant clinical nuances.^11^ The purpose in each separate clinical case was to elicit a treatment decision for the hypothetical patient. The question wording was, “Would you recommend that [patient] continue or discontinue [treatment]?” The 4 response options were “definitely continue,” “tend to continue,” “tend to discontinue,” and “definitely discontinue.”

Each scenario appeared in 1 of 2 different versions, termed the *improved* version and the *unchanged* version. The improved version described a marginal improvement in symptoms after initial treatment. The unchanged version described unchanged symptoms after initial treatment. No scenario described a worsening of symptoms that might have otherwise justified discontinuing treatment. In all other respects, the 2 versions were identical, involved the same hypothetical patient, contained a single question, and were randomly assigned. Given the information about symptoms following treatment, participants then decided whether to recommend continuing or discontinuing the treatment. For a few scenarios, we added a third, untested version that gauged participants’ decisions to recommend the treatment before it had been tested.

Several survey features were designed to minimize bias. First, each scenario was conceived on the basis of direct clinical experience to make the case meaningful, original, clear, realistic, and relevant. Second, no identifying demographic information on participants was collected to maintain confidentiality and reduce respondent workload. Third, the scenarios all described a patient who had few comorbidities, thus minimizing complexity and reducing the need for medical expertise. Fourth, each respondent saw only one version of one scenario to reduce boredom or carry-over artifacts. Finally, scenarios were designed to examine widely different common clinical problems, allowing for potential generalizability.

### Participants

We surveyed different participant groups in North America. The largest group was community participants recruited through the Prolific Survey platform.^12^ The second group consisted of active community pharmacists who were contacted in person while in their place of work at off-peak regular business hours. The intent was to elicit judgments from potential patients and from health care professionals who were experienced, informed, engaged, and commonly interacting with patients. Community participants were surveyed in the early winter months of 2023 and 2024, and pharmacists were surveyed in the summer months of 2023.

All participants completed the survey anonymously with a planned time-to-completion of less than 2 minutes. Participants were blinded to the hypothesis, given a single scenario, and unaware of alternative versions. Community participants received the survey online and were compensated at the standard internet platform rate ($15 per hour). Further background details on the community participants appear in the eAppendix in Supplement 1. The introductory script for the pharmacists was, “I would like to talk with a pharmacist,” with additional contingent replies formulated in advance using methods for secret shopper science.^13^ Pharmacists received no incentives (aside from a gracious thank-you).

The study was approved by the Research Ethics Board of Princeton University and the Sunnybrook Research Institute. The approval included a waiver for signed consent, with willingness to participate expressed by continued activity on the platform (and the freedom to discontinue at any point). The pharmacists were contacted in person and later received a debriefing letter that provided an explanation of the research, a statement of what was done, an expression of gratitude, an opportunity for discussion, reassurances of anonymization, contact details for concerns, and the option to withdraw (none subsequently withdrew). This study follows the American Association for Public Opinion Research (AAPOR) reporting guideline.

No other groups were involved in the study, and no participants were excluded from analysis. Participants and the public were not involved in the research design or study reporting.

### Statistical Analysis

The primary analysis compared responses to the 2 versions of each scenario, expressed as the proportion of respondents who recommended (tend to or definitely) continuing the treatment. Statistical analyses used the χ^2^ test and an odds ratio (OR) estimate for a consistent measure of effect size (analyses using the Mann-Whitney test to account for the full nonparametric distribution yielded similar results and appear in the eAppendix in Supplement 1). Sample size calculations were designed to provide 80% power (β = 0.20) for detecting an effect size of at least 15% (baseline of 10%, increase to 25%). The analytic plan was specified in advance, each scenario was considered an independent test, all *P* values were 2-tailed, and OR estimates were accompanied by 95% CIs.

## Results

In total, 1497 community members (mean [SD] age, 38.1 [12.5] years; 663 female [55.3%]) were contacted. Additional demographic data for community members are shown in the eAppendix in Supplement 1. One hundred pharmacists were also contacted.

### Antibiotic Scenario

The first case involved using antibiotic treatment contrary to medical science. The improved and unchanged versions were identical except the bold text (improved version) was replaced by the bold text in brackets (unchanged version): “JL is a 35-year-old schoolteacher. She has a sore throat and starts an antibiotic treatment from an unused prescription offered by a friend. According to medical science, however, misusing antibiotics might eventually cause resistant organisms. The next day JL feels **better [unchanged].** Would you recommend she continue or discontinue the antibiotic?”

A total of 300 individuals responded, of whom 150 received the improved version and 150 received the unchanged version. As hypothesized, more recommended continuing the antibiotic after symptoms subjectively improved than when symptoms were unchanged (67 respondents [45%] vs 25 respondents [17%]). This discrepancy equaled a moderate relative increase in continued treatment (OR, 3.98; 95% CI, 2.33-6.78; *P* < .001) (Table).

**Table.** Summary of Main Results

Scenario	Initial response to treatment, respondents, No./total No. (%)		OR (95% CI)^a^
	Improved	Unchanged	
Antibiotic	67/150 (45)	25/150 (17)	3.98 (2.33-6.78)
Sugar	83/100 (83)	17/100 (17)	22.77 (10.99-47.18)
Acupuncture	87/100 (87)	29/98 (28)	15.27 (7.46-31.27)
Bracelet	78/100 (78)	25/99 (25)	16.19 (5.32-19.52)
Baldness	129/200 (65)	14/200 (7)	23.30 (12.69-42.16)
Fatigue	80/100 (80)	33/100 (33)	7.91 (4.18-14.97)
Replication^b^	36/44 (82)	35/56 (63)	2.60 (1.04-6.52)

Abbreviation: OR, odds ratio.

^a^ Based on dichotomize distribution of responses.

^b^ Scenario assigned to health care workers.

### Sugar Scenario

This case involved considering an unproven sugar supplement for insomnia. The improved and unchanged versions were identical except the bold text was replaced by the bold text in brackets: “GD is a 45-year-old administrator. She has irregular insomnia and starts a sugar powder supplement in the hopes of getting better sleep. The next week her sleep is **better [unchanged].** Would you recommend she continue or discontinue the sugar powder supplement?”

A total of 200 individuals responded, of whom 100 received the improved version and 100 the unchanged version. As hypothesized, more recommended continuing the sugar after symptoms improved than when symptoms were unchanged (83 respondents [83%] vs 17 respondents [17%]). This discrepancy amounted to a large increase in continued treatment (OR, 22.77; 95% CI, 10.99-47.18; *P* < .001) (Table).

### Acupuncture Scenario

This case involved a family member suggesting an alternative remedy to a young woman with neck pain. The improved and unchanged versions were identical except the bold text was replaced by the bold text in brackets: “KA is a 30-year-old flight attendant. She feels intermittent neck pain and starts acupuncture for relief (suggested by her grandmother). The next week she feels **better [unchanged].** Would you recommend she continue or discontinue the acupuncture?”

A total of 198 individuals responded, of whom 100 received the improved version and 98 the unchanged version. As hypothesized, more recommended continuing the acupuncture after symptoms improved than when symptoms were unchanged (87 respondents [87%] vs 29 respondents [28%]). This discrepancy amounted to a large increase in continued treatment (OR, 15.27; 95% CI, 7.46-31.27; *P* < .001) (Table).

### Bracelet Scenario

This case involved the role of advertising of commercial products promoted for wrist pain. The improved and unchanged versions were identical except the bold text was replaced by the bold text in brackets: “MB is a 45-year-old accountant. She feels intermittent wrist pain and starts wearing a copper bracelet for pain relief (advertised in a magazine). The next week she feels **better [unchanged].** Would you recommend she continue or discontinue the copper bracelet?”

A total of 199 individuals responded, of whom 100 received the improved version and 99 the unchanged version. As hypothesized, more recommended continuing the copper bracelet after symptoms improved than when symptoms were unchanged (78 respondents [78%] vs 25 respondents [25%]). This discrepancy amounted to a large increase (OR, 16.19; 95% CI, 5.32-19.52; *P* < .001) (Table).

### Shampoo Scenario

This case involved an outlandish treatment directly contrary to research evidence. The improved and unchanged versions were identical except the bold text was replaced by the bold text in brackets: “DK is a 75-year-old retired professor. He is going bald and starts applying horse shampoo once per day. However, researchers typically agree horse shampoo does not promote hair growth. A couple of weeks later his hair seems **better [unchanged].** Suppose he is undecided and asks you, would you recommend DK continue or discontinue the horse shampoo?”

A total of 400 individuals responded, of whom 200 received the improved version and 200 the unchanged version. As hypothesized, more recommended continuing the horse shampoo after symptoms improved than when symptoms were unchanged (129 respondents [65%] vs 14 respondents [7%]). This discrepancy amounted to a very large increase in continued treatment (OR, 23.30; 95% CI, 12.69-42.16; *P* < .001) (Table).

### Vitamin Supplement Scenario

This case involved considering a vitamin supplement for fatigue. The improved and unchanged versions were identical except the bold text was replaced by the bold text in brackets: “WS is a 30-year-old hospital nurse. He feels tired and starts a Vitamin B12 supplement for energy (despite normal B12 levels). The next week he feels **better [unchanged]**. Would you recommend he continue or discontinue the Vitamin B12 supplement?”

A total of 200 individuals responded, of whom 100 received the improved version and 100 the unchanged version. Despite the normal baseline B_12_ level, many respondents recommended continuing the vitamin B_12_ supplement. As hypothesized, more recommended continuing after symptoms improved than when symptoms were unchanged (80 respondents [80%] vs 33 respondents [33%]). This discrepancy equaled a substantial relative increase in treatment (OR, 7.91; 95% CI, 4.18-14.97; *P* < .001) (Table).

### Replication Study

The replication study involved a situation similar to the preceding fatigue scenario, this time enacted by a secret shopper visiting different pharmacies and asking the pharmacists for advice. The improved and unchanged versions were identical except the bold text was replaced by the bold text in brackets. The wording was, “Hi, I am wondering if I can talk with a pharmacist? [Wait for pharmacist.] Last week I was feeling tired and started taking a Vitamin B12 supplement for energy. Now this week I feel **better [unchanged]**. Do you think I should continue or discontinue it?”

A total of 100 pharmacists were contacted, of whom 44 and 56 received the improved and unchanged versions, respectively. More pharmacists recommended continuing the treatment after symptoms improved than when the symptoms were unchanged (36 pharmacists [82%] vs 35 pharmacists [63%]). This discrepancy equaled a modest significant relative increase in recommended treatment (OR, 2.60; 95% CI, 1.04-6.52; *P* = .03) (Table).

## Discussion

### Overview

Through scenarios involving diverse conditions, this survey study identified a persistent tendency toward continuing a treatment when initial care was followed by some subjective improvement. Although a modicum of enthusiasm might be expected, the substantially increased preferences for continuation are unwarranted because the improvement is not necessarily caused by the treatment. The post hoc bias might both increase a patient’s willingness to continue and also to recommend the treatment to others. The same error might also lead to undertreatment if a random adverse event leads to a worsening of symptoms.^14^ These strong patient intuitions are misplaced because the situation is not a randomized trial sufficient to prove effectiveness.^15^

### Selective Justifications

A reliance on the post hoc bias might be an expedient compromise in some cases. Sometimes the mistake may be harmless when undue treatment is relatively safe and entirely affordable. It can also be innocuous for patients with self-limited diseases when no other critical diagnoses or interventions are missed. In addition, a post hoc bias might solidify a doctor-patient relationship by exemplifying attention, compassion, and continuity of care. Continuing treatment after an improvement also aligns with the traditional aphorism, “don’t mess with success.” Abiding by a modicum of post hoc bias is sometimes the most straightforward position, whereas the effort to resolve the misunderstanding is not always worth the trouble.

Continuing a treatment is, of course, sometimes also warranted. Some treatments are definitive, such as bullet extraction for saving a patient from a gunshot wound. In other cases, the reasoning is correct owing to enormous effectiveness, such as antiviral drugs for HIV infection. Some treatments are convincing with long-term follow-up (eg, hip replacement for advanced osteoarthritis), and other treatments are confined to extreme cases (eg, cardiac defibrillation for a cardiac arrest). Many metabolic disorders can be cured, such as thyroid replacement for hypothyroidism. Intuitive judgments about effectiveness, however, can be mistaken when they overlook the contribution of rest, attention, time, or other factors irrelevant to the treatment.

### Fundamental Misconception

Our study highlights a core weakness around intuitions in health care. The simple logic of cause-and-effect reasoning implies that action leads to outcome; however, human biology is complex, with many active factors influencing a single patient. These factors include surrounding supportive care (medications, fluids, diet changes, and physiotherapy), as well as unobserved cointerventions (cessation of adverse behaviors, separation from harmful environments, physiologic homeostasis, and placebo effects). Furthermore, simple regression to the mean can lead to improvement, since people often resort to dubious treatments when things get worse.^16^ Simplifying patient outcomes to a single medical intervention is arguably the fundamental fallacy emblematic of reductionist perspectives.^17^

Clinicians often acknowledge complexity in the aftermath of failure—namely, patient outcomes are uncertain. That same humility can feel less likely in the aftermath of a success where adherence to scientific discipline can be difficult following a treatment initiated by the responsible clinician.^18^ Personal motivations might further reinforce undue confidence after an improvement since clinicians want to believe their work makes a positive difference.^19^ Faulty attribution may be further exacerbated when the clinician interacts with a grateful patient, appreciative family, and impressed colleagues. In short, the system surrounding care helps perpetuate the post hoc bias.

### Post Hoc Discontinuation

Our study focused on the tendency to continue a dubious treatment, whereas post hoc bias might also lead to prematurely stopping an appropriate treatment. Specifically, we examined an additional, untested version of some scenarios where a treatment was under consideration but not yet tried. In line with post hoc bias, we found reduced recommendations for treatment after no improvement occurred compared with before the treatment was initiated. In the acupuncture scenario, for example, few recommended continued treatment in the unchanged version, despite most recommending treatment in the untested version (eAppendix in Supplement 1). Similarly, in the vitamin supplement scenario, fewer participants recommended treatment when no immediate change occurred compared with the untested version. Apparently, enthusiasm can quickly diminish after no signs of improvement.

A tendency to prematurely stop a treatment is another facet of post hoc bias that serves to illuminate an important point. In some cases, a placebo effect might be sufficient justification to continue a harmless treatment, and a nocebo effect might justify discontinuation after an adverse event.^20^ It is noteworthy, however, that situations such as the antibiotic case and the sugar supplement case raise adverse consequences beyond the putative benefits of a placebo effect. Furthermore, neither a presumed placebo effect nor a nocebo effect can explain the post hoc tendency to discontinue treatment after no change. The post hoc tendencies we observe following no change in symptoms serve to illustrate that placebo considerations cannot explain post hoc bias more generally.

### Limitations

Our study has several limitations that merit mention. We examined simple decisions, whereas medicine is often more complicated. The study involved brief scenarios with time for thought, whereas clinical settings often involve distractions, uncertainties, and conflicting priorities. We did not collect baseline characteristics on participants or conduct interviews inquiring about their reasoning. Future research could also test whether post hoc bias can be mitigated by patient education or whether it extends more broadly (eg, when symptoms worsen or treatment effects are delayed). To be sure, our randomized design renders the results difficult to attribute to atypical respondents, sloppy responses, strategic ploys, fallible information, or random chance.

We tested a small number of scenarios, whereas post hoc bias might extend to a variety of faulty beliefs that worsen with experience. Similar to other pitfalls in reasoning, post hoc bias may be more easily detected in others than in oneself and might otherwise persist despite personal experience.^21^ Years of practicing bloodletting in the history of medicine, for example, led to some patients improving by chance and to reinforcing anecdotes of successful treatment.^22^ Similarly, modern care abounds with patients who blame vaccines for subsequent unrelated illnesses.^23,24^ Interestingly, the opposite of post hoc bias might occur in preventive medicine, where the absence of an event is often unrecognized and rarely attributed to an earlier intervention.

## Conclusions

We found that a marginal improvement in symptoms tends to encourage patients to continue a treatment even when the change might be coincidental. Similarly, a lack of improvement can lead patients to discontinue treatment too soon. Akin to other heuristics, this intuition is sometimes appropriate and not always mistaken. Reliance on post hoc reasoning is logical when a diagnosis is certain and treatment infallible; otherwise, post hoc bias can contribute to complacency, discourage the search for alternatives, and lead to shortfalls in care. An awareness of post hoc bias will not make it disappear; however, it might help if clinicians reminded patients that symptomatic improvements still merit considering alternative explanations.

## Notes

Supplement 1. **eAppendix.** Technical appendix

Supplement 2.
Data Sharing Statement
