# Supplementary material for: Post Hoc Bias in Treatment Decisions
Source: JAMA Netw Open. 2024 Sep 4;7(9):e2431123. doi: 10.1001/jamanetworkopen.2024.31123 (PMC11375477; doi:10.1001/jamanetworkopen.2024.31123)
Supplement: Supplement 1. — eAppendix. Technical appendix [file jamanetwopen-e2431123-s001.pdf]

Supplemental Online Content

Redelmeier DA, Shafir E. Post hoc bias in treatment decisions. *JAMA Netw Open*. 2024;7(9):e2431123. doi:10.1001/jamanetworkopen.2024.31123

eAppendix. Technical appendix

This supplemental material has been provided by the authors to give readers additional information about their work.

© 2024 Redelmeier DA et al. *JAMA Network Open.*

eAppendix. Technical Appendix

This appendix provides further background information on the community participants, the full text details for all scenarios, and the response data for complete distributions of aggregated data.

Community Participants

<u>Prolific Survey Platform</u>

Online crowdsourcing is a recent innovation for conducting studies in experimental psychology. Similar to research using laboratory conditions, online crowdsourcing can include large sample sizes, exact randomization procedures, double-blinding of participants, and rigorous statistical analysis. Unlike research in laboratory conditions, online crowdsourcing does not require in-person contact; is marginally biased toward younger, female, and more educated participants; and is more appropriate for short simple surveys. Some past studies suggest that online experiments can yield similar results to laboratory experiments (DOI: 10.1016/j.chb.2013.05.009).

Prolific is one of several platform for online crowdsourcing. Alternatives include MTurk, SurveyMonkey, Qualtrics, CloudResearch, CrowdFlower, and others (DOI: 10.1016/j.jesp.2017.01.006). Past studies suggest the data can be as reliable as those from traditional methods (DOI: 10.1177/1745691610393980). Major limitations focus on tasks that require sustained attention, comprehension of complex instructions, participants completing multiple trials, or millisecond accuracy for response recording (DOI: 10.1371/journal.pone.0057410). Our community sample was based on the Prolific platform with details available online (https://researcher-help.prolific.com/hc/en-gb).

The Prolific platform is based in the United Kingdom, reflects participants primarily from English-speaking countries, and has strengths for online crowdsourcing. The sample is arguably more demographically diverse than samples of university undergraduates. The available pool is substantial so a sample size of 100 can often be recruited in a single day. Data quality is regularly checked for comprehension, attention, and dishonesty through internal algorithms for quality control. Early studies suggest participants sometimes also enjoy secondary benefits including self-improvement or emotional gains beyond the financial compensation (DOI: 10.1109/HICSS.2015.98).

Baseline Demographics for Current Study (n = 1,199 of 1,497)

Sex

Sex	Count	Percent
DATA_EXPIRED	2	0.1668057
Female	663	55.2960801
Male	530	44.2035029
Prefer not to say	4	0.3336113

Age

Mean	Median	Standard Deviation
38.06505	35	12.52166

Ethnicity

Ethnicity	Count	Percent
Asian	126	10.508757
Black	110	9.174312
DATA_EXPIRED	24	2.001668
Mixed	85	7.089241
Other	36	3.002502
White	818	68.223520

Nationality

Country	Count	Percent
United States	1,199	100.00

© 2024 Redelmeier DA et al. *JAMA Network Open.*

Country of Birth

Country of Birth
1. Azerbaijan<br>2. Bangladesh<br>3. Canada<br>**4. China –** 0.42%<br>5. Cote d'Ivoire<br>6. DATA_EXPIRED **–** 0.67%<br>7. Dominican Republic<br>8. Finland<br>9. France<br>10. Germany<br>11. Ghana<br>12. Greece<br>13. Guam<br>14. Guyana<br>15. Haiti<br>16. Honduras<br>**17. India –** 0.50%<br>18. Indonesia<br>19. Ireland<br>20. Italy<br>21. Jamaica<br>22. Japan<br>23. Korea<br>24. Lebanon<br>25. Malaysia<br>26. Mexico<br>**27. Nigeria –** 0.92%<br>28. Pakistan<br>29. Palestinian Territory<br>30. Peru<br>**31. Philippines –** 0.33%<br>32. Portugal<br>33. Puerto Rico<br>34. South Africa<br>35. Taiwan<br>36. Thailand<br>37. Turkey<br>38. United Kingdom<br>**39. United States –** 92.41% (1108)<br>40. United States Minor Outlying Islands<br>41. Venezuela, Bolivarian Republic of<br>42. Vietnam

© 2024 Redelmeier DA et al. *JAMA Network Open.*

Antibiotic Scenario

Improved Version (n = 150)

JL is a 35-year-old schoolteacher. She has a sore throat and starts an antibiotic treatment from an unused prescription offered by a friend. According to medical science, however, misusing antibiotics might eventually cause resistant organisms. The next day JL feels better. Would you recommend she continue or discontinue the antibiotic?

Definitely Continue	Tend to Continue	Tend to Discontinue	Definitely Discontinue
22	45	38	45

Unchanged Version (n = 150)

JL is a 35-year-old schoolteacher. She has a sore throat and starts an antibiotic treatment from an unused prescription offered by a friend. According to medical science, however, misusing antibiotics might eventually cause resistant organisms. The next day JL feels unchanged. Would you recommend she continue or discontinue the antibiotic?

Definitely Continue	Tend to Continue	Tend to Discontinue	Definitely Discontinue
8	17	36	89

Untested Version (n = 150)

JL is a 35-year-old school teacher. She has a sore throat and considers starting an antibiotic treatment from an unused prescription offered by a friend. According to medical science, however, misusing antibiotics might eventually cause resistant organisms. Would you recommend she start or not start the antibiotic?

Definitely Start	Tend to Start	Tend to Not Start	Definitely Not Start
1	28	54	67

© 2024 Redelmeier DA et al. *JAMA Network Open.*

Sugar Scenario

Improved Version (n = 100)

GD is a 45-year-old administrator. She has irregular insomnia and starts a sugar powder supplement in the hopes of getting better sleep. The next week her sleep is better. Would you recommend she continue or discontinue the sugar powder supplement?

Definitely Continue	Tend to Continue	Tend to Discontinue	Definitely Discontinue
20	63	11	6

Unchanged Version (n = 100)

GD is a 45-year-old administrator. She has irregular insomnia and starts a sugar powder supplement in the hopes of getting better sleep. The next week her sleep is unchanged. Would you recommend she continue or discontinue the sugar powder supplement?

Definitely Continue	Tend to Continue	Tend to Discontinue	Definitely Discontinue
1	16	41	42

Untested Version (n = 100)

GD is a 45-year-old administrator. She has irregular insomnia and considers starting a sugar powder supplement in the hopes of getting better sleep. Would you recommend she start or not start the sugar powder supplement?

Definitely Start	Tend to Start	Tend to Not Start	Definitely Not Start
3	17	41	38

© 2024 Redelmeier DA et al. *JAMA Network Open.*

Acupuncture Scenario

Improved Version (n = 100)
KA is a 30-year-old flight attendant. She feels intermittent neck pain and starts acupuncture for relief (suggested by her grandmother). The next week she feels better. Would you recommend she continue or discontinue the acupuncture?

Definitely Continue	Tend to Continue	Tend to Discontinue	Definitely Discontinue
31	56	11	2

Unchanged Version (n = 98)
KA is a 30-year-old flight attendant. She feels intermittent neck pain and starts acupuncture for relief (suggested by her grandmother). The next week she feels unchanged. Would you recommend she continue or discontinue the acupuncture?

Definitely Continue	Tend to Continue	Tend to Discontinue	Definitely Discontinue
2	27	46	23

Untested Version (n = 100)
KA is a 30-year-old flight attendant. She feels intermittent neck pain and considers starting acupuncture for relief (suggested by her grandmother). Would you recommend she start or not start acupuncture?

Definitely Start	Tend to Start	Tend to Not Start	Definitely Start
18	57	19	6

© 2024 Redelmeier DA et al. *JAMA Network Open.*

Bracelet Scenario

Improved Version (n = 100)
MB is a 45-year-old accountant. She feels intermittent wrist pain and starts wearing a copper bracelet for pain relief (advertised in a magazine). The next week she feels better. Would you recommend she continue or discontinue the copper bracelet?

Definitely Continue	Tend to Continue	Tend to Discontinue	Definitely Discontinue
22	56	17	5

Unchanged Version (n = 99)
MB is a 45-year-old accountant. She feels intermittent wrist pain and starts wearing a copper bracelet for pain relief (advertised in a magazine). The next week she feels better unchanged. Would you recommend she continue or discontinue the copper bracelet?

Definitely Continue	Tend to Continue	Tend to Discontinue	Definitely Discontinue
4	21	47	27

Untested Version (n = 100)
MB is a 45-year-old accountant. She feels intermittent wrist pain and considers wearing a copper bracelet for pain relief (advertised in a magazine). Would you recommend she start or not try the copper bracelet?

Definitely Start	Tend to Start	Tend to Not Start	Definitely Not Start
2	41	30	27

© 2024 Redelmeier DA et al. *JAMA Network Open.*

Shampoo Scenario (WITHOUT DOUBTS)

<u>Improved Version (n = 150)</u>
DK is an 75-year-old retired professor. He is going bald and starts applying horse shampoo once per day. However, researchers typically agree horse shampoo does not promote hair growth. A couple of weeks later his hair seems better. Would you recommend DK continue or discontinue the horse shampoo?

Definitely Continue	Tend to Continue	Tend to Discontinue	Definitely Discontinue
10	83	34	23

<u>Unchanged Version (n = 150)</u>
DK is an 75-year-old retired professor. He is going bald and starts applying horse shampoo once per day. However, researchers typically agree horse shampoo does not promote hair growth. A couple of weeks later his hair seems unchanged. Would you recommend DK continue or discontinue the horse shampoo?

Definitely Continue	Tend to Continue	Tend to Discontinue	Definitely Discontinue
0	6	41	103

<u>Untested Version (n = 150)</u>
DK is an 75-year-old retired professor. He is going bald and considers applying horse shampoo once per day. However, researchers typically agree horse shampoo does not promote hair growth. Would you recommend DK start or not start the horse shampoo?

Definitely Start	Tend to Start	Tend to Not Start	Definitely Not Start
2	14	35	99

© 2024 Redelmeier DA et al. *JAMA Network Open.*

Shampoo Scenario (WITH DOUBTS)

Improved Version (n = 200)
DK is an 75-year-old retired professor. He is going bald and starts applying horse shampoo once per day. However, researchers typically agree horse shampoo does not promote hair growth. A couple of weeks later his hair seems better. Suppose he is undecided and asks you, would you recommend DK continue or discontinue the horse shampoo?

Definitely Continue	Tend to Continue	Tend to Discontinue	Definitely Discontinue
18	111	52	19

Unchanged Version (n = 200)
DK is an 75-year-old retired professor. He is going bald and starts applying horse shampoo once per day. However, researchers typically agree horse shampoo does not promote hair growth. A couple of weeks later his hair seems unchanged. Suppose he is undecided and asks you, would you recommend DK continue or discontinue the horse shampoo?

Definitely Continue	Tend to Continue	Tend to Discontinue	Definitely Discontinue
5	9	X44X	142

© 2024 Redelmeier DA et al. *JAMA Network Open.*

Vitamin Supplement Scenario (AS SURVEY)

Improved Version (n = 100)

WS is a 30-year-old hospital nurse. He feels tired and starts a Vitamin B12 supplement for energy (despite normal B12 levels). The next week he feels better. Would you recommend he continue or discontinue the Vitamin B12 supplement?

Definitely Continue	Tend to Continue	Tend to Discontinue	Definitely Discontinue
26	54	16	4

Unchanged Version (n = 100)

WS is a 30-year-old hospital nurse. He feels tired and starts a Vitamin B12 supplement for energy (despite normal B12 levels). The next week he feels unchanged. Would you recommend he continue or discontinue the Vitamin B12 supplement?

Definitely Continue	Tend to Continue	Tend to Discontinue	Definitely Discontinue
5	28	46	21

Untested Version (n = 100)

WS is a 30-year-old hospital nurse. He feels tired and considers starting a Vitamin B12 supplement for energy (despite normal B12 levels). Would you recommend he start or not start the Vitamin B12 supplement?

Definitely Start	Tend to Start	Tend to Not Start	Definitely Not Start
13	39	37	11

© 2024 Redelmeier DA et al. *JAMA Network Open.*

Vitamin Supplement Scenario (AS VALIDATION)

Improved Version (n = 44)

Hi, I am wondering if I can talk with a pharmacist? {… wait for pharmacist …} Last week I was feeling tired and started taking a Vitamin B12 supplement for energy. Now this week I feel better. Do you think I should continue or discontinue it?

Continue	Discontinue
36	8

Unchanged Version (n = 56)

Hi, I am wondering if I can talk with a pharmacist? {… wait for pharmacist …} Last week I was feeling tired and started taking a Vitamin B12 supplement for energy. Now this week I feel unchanged. Do you think I should continue or discontinue it?

Continue	Discontinue
35	21

Contingency Scripts

Any other health changes besides fatigue? “Nothing else that I’ve noticed.”
Have you seen a physician about this? “I don’t think I need a physician.”
Did a physician tell you to take B12? “No, I don’t have a physician.”
How did you first get the idea to try B12? “My mom suggested it.”
Can I see the B12 you’ve been taking? “Sure, here’s the bottle.”
Would you like to buy some B12? “Sure, I will buy it here when I run out.”
Are you a regular at our pharmacy? “No, I am only here for school.”
Do you have a special diet? “I eat everything.”

© 2024 Redelmeier DA et al. *JAMA Network Open.*
