# Supplementary material for: Post Hoc Bias in Treatment Decisions
Source: JAMA Netw Open. 2024 Sep 4;7(9):e2431123. doi: 10.1001/jamanetworkopen.2024.31123 (PMC11375477; doi:10.1001/jamanetworkopen.2024.31123)
Supplement: Supplement 2. — Data Sharing Statement [file jamanetwopen-e2431123-s002.pdf]

Data Sharing Statement

Redelmeier. Post Hoc Bias in Treatment Decisions. *JAMA Netw Open*. Published September 04, 2024. doi:10.1001/jamanetworkopen.2024.31123

Data
Data available: Yes
Data types: Deidentified participant data
How to access data: Aggregated data shown in accompanying appendix
When available: With publication

Supporting Documents
Document types: None

Additional Information
Who can access the data: Aggregated data shown in accompanying appendix
Types of analyses: Any purpose
Mechanisms of data availability: No support
Any additional restrictions: No restrictions
